# The clinical value of miR‐193a‐3p in non‐small cell lung cancer and its potential molecular mechanism explored *in silico* using RNA‐sequencing and microarray data

**DOI:** 10.1002/2211-5463.12354

**Published:** 2018-01-04

**Authors:** Xiang Gao, Rui‐xue Tang, Qiong‐ni Xie, Jia‐ying Lin, Hong‐lan Shi, Gang Chen, Zu‐yun Li

**Affiliations:** ^1^ Department of Medical Oncology First Affiliated Hospital of Guangxi Medical University Nanning China; ^2^ Department of Pathology First Affiliated Hospital of Guangxi Medical University Nanning China

**Keywords:** GEO, miR‐193a‐3p, non‐small cell lung cancer, pathways, target genes, TCGA

## Abstract

miR‐193a‐3p is a tumor‐related miRNA playing an essential role in tumorigenesis and progression of non‐small cell lung cancer (NSCLC). The objective of the present study was to investigate the relationship between miR‐193a‐3p expression and clinical value and to further explore the potential signaling of miR‐193a‐3p in the carcinogenesis of NSCLC. RNA‐sequencing and microarray data were collected from the databases GEO, ArrayExpress and The Cancer Genome Atlas (TCGA). Furthermore, *in silico* assessments were performed to analyze the prospective pathways and networks of the target genes of miR‐193a‐3p. In total, 453 cases of NSCLC patients and 476 normal controls were included in blood samples, while 920 cases of NSCLC patients and 406 normal controls were included in tissue samples. The pooled positive likelihood ratio, the pooled negative likelihood ratio and the pooled diagnostic odds ratio were calculated to reflect the diagnostic value of miR‐193a‐3p in blood and tissue samples. Moreover, the areas under the curve of the summary receiver operating characteristic curve of blood and tissue were 0.64 and 0.79, respectively. In addition, we found a lower level of miR‐193a in NSCLC tissues than in non‐cancerous controls based on TCGA. A gene ontology (GO) enrichment analysis demonstrated that miR‐193a‐3p could be related to key signaling pathways in NSCLC. Also, several vital pathways were illustrated by KEGG. Lower expression of miR‐193a‐3p in tissue samples of NSCLC may be associated with tumorigenesis and be a predictor of deterioration of NSCLC patients, and pathway analysis revealed crucial signaling pathways correlated with the incidence and progress of NSCLC.

**Abbreviations**
AUC: area under the curve
CI: confidence interval
DAVID: Database for Annotation, Visualization and Integrated Discovery
GO: Gene Ontology
KEGG: Kyoto Encyclopedia of Genes and Genomes
LR: likelihood ratio
LUAD: lung adenocarcinoma
LUSC: lung squamous cell carcinoma
miRNA: microRNA
NSCLC: non‐small cell lung cancer
OR: odds ratio
PPI: protein–protein interaction
PT: paracancer tissue
ROC: receiver operating characteristic
SMD: standard mean difference
SROC: summary receiver operating characteristic
TCGA: The Cancer Genome Atlas
TNM: tumor, node and metastasis

Lung cancer is one of the most severe malignancies threatening human health. According to the latest cancer statistics published in 2017, it remained the main cause of death in both men and women 1. In China, lung cancer was also the most frequent cancer and the top cause of cancer death in 2015 2. The latest data provided by The National Central Cancer Registry of China revealed that non‐small cell lung cancer (NSCLC) accounted for ~80–85% of newly diagnosed cases of lung cancer, and the 5‐year survival rate of NSCLC was approximately 11% 3. Currently, standard diagnostic approaches mainly include radiography, computed tomography, bronchial needle aspiration biopsy guided by ultrasound and detection of bronchial lavage tumor markers. Despite the advantages of these available diagnostic strategies, the lack of sufficient specificity and sensitivity creates a challenge for the identification of lung cancer at an early stage 4. Tumor biomarkers have attracted great attention in the research area of lung cancer, since this cancer's occurrence is a result of a long‐term process presenting with the change of a normal cell to a malignant one, which includes gradual genetic alterations 5. Many lung cancer‐related oncogenes, as well as tumor suppressors, have been identified, and aberrations of several signaling pathways have also been discovered 6. Nevertheless, the specific molecular mechanism for the occurrence of lung cancer still remains uncertain. Recent studies have confirmed the correlation between lung cancer and non‐coding RNAs, among which microRNAs (miRNAs) account for a large proportion.

miRNAs are a type of naturally occurring, small non‐coding RNA molecule with approximately 21–25 nucleotides 7. Recently, an increasing amount of research has shown that abnormally expressed miRNAs participating in the incidence and development of malignant tumors may become a new type of tumor marker 8. miRNAs can control numerous biological pathways driving tumor behavior by targeting and controlling gene expression in lung cancer 9, 10. The characteristics of miRNAs modulating gene networks and corresponding biological pathways have provided new hope for diagnosing and guiding novel therapeutic decisions for lung cancer patients 10. However, numerous miRNAs need to be identified and their potential molecular mechanisms still remain to be further determined.

miR‐193a‐3p is one of the cancer‐related miRNAs. It has been found that up‐regulated miR‐193a‐3p functions as an oncogene in esophageal squamous cell carcinoma, modulating proliferation, migration and apoptosis 11. Aberrant regulation of miR‐193a‐3p has also been found in the development of other types of cancer, such as prostate cancer, breast cancer, head and neck squamous cell carcinomas, and colorectal cancer 12, 13, 14, 15. Previously, we reported that miR‐193a‐3p might be a tumor suppressor in hepatocellular carcinoma. Besides, the expression level of serum miR‐193a‐3p combined with alpha‐fetoprotein and ultrasound could assist in the diagnosis of hepatocellular carcinoma at an early stage 16. Our previous work using quantitative real‐time polymerase chain reaction also demonstrated that the miR‐193a‐3p level in NSCLC tissue samples was significantly down‐regulated compared with that in non‐tumorous lung tissues 17. Several other reports also determined the level of miR‐193a‐3p in lung cancer tissues, but the sample size varied and the results were inconsistent. However, no study has mined the public high‐throughput data of RNA‐sequencing and microarray to explore the clinical role of miR‐193a‐3p in lung cancer.

The function and molecular mechanisms of miR‐193a‐3p have been explored in a few diseases. For instance, in human osteosarcoma, miR‐193a‐3p functioned as a suppressor by suppressing the signaling pathway of Rab27B and serine racemase 18, 19. Similarly, the suppressive influence of miR‐193a‐3p on growth was related to the reduction of *MCL1* expression in malignant pleural mesothelioma 20. In NSCLC, only three targets have been confirmed, including *ERBB4*,* S6K2* and *KRas* 19, 21, 22. Nevertheless, other specific regulatory mechanisms of miR‐193a‐3p in lung cancer are still uncertain, due to the diversity of target genes regulated by a single miRNA. Hence, the objective of the present study was to evaluate the association between miR‐193a‐3p level and the development of NSCLC using public high‐throughput data including RNA‐sequencing, microarray and all available published documents. Further, we also explored the potential signaling of miR‐193a‐3p in the carcinogenesis of NSCLC via *in silico* approaches, such as Gene Ontology (GO), Kyoto Encyclopedia of Genes and Genomes (KEGG) and protein–protein interaction (PPI) pathway analyses.

## Materials and methods

### Data acquisition in GEO and ArrayExpress datasets

#### Search strategy and inclusion criteria

We searched the NSCLC‐related miRNA microarray or RNA‐sequencing data from the National Center of Biotechnology Information (NCBI) Gene Expression Omnibus (GEO; http://www.ncbi.nlm.nih.gov/geo/) and ArrayExpress to 1 July 2017.

The search strategy was as follows: (lung OR pulmonary OR respiratory OR bronchi OR bronchioles OR alveoli OR pneumocytes OR ‘air way’) AND (cancer OR carcinoma OR tumor OR neoplas* OR malignan* OR adenocarcinoma) AND (MicroRNA OR miRNA OR ‘Micro RNA’ OR ‘Small Temporal RNA’ OR ‘non‐coding RNA’ OR ncRNA OR ‘small RNA’).

The inclusion criteria of eligible datasets were as follows. Firstly, the experimental group should be NSCLC patients and the control group should be non‐cancerous individuals. Secondly, the minimum number of samples for each group should be 30. Thirdly, the raw miRNAs expression data from profiling of experimental and control groups should be available or calculable, which provided the expression level of miR‐193a‐3p. Fourthly, only human samples were included. Fifthly, both tissue and peripheral blood samples from NSCLC were included.

#### Statistical analysis

Firstly, the expression level of miR‐193a‐3p was extracted from each microarray. Student's paired or unpaired *t* test was used to assess the difference of miR‐193a‐3p level between different groups with spss statistics v. 23.0 (IBM Corp., Armonk, NY, USA). Receiver operating characteristic (ROC) curve analyses were used to assess the diagnostic significance of miR‐193a‐3p in NSCLC. *P* < 0.05 was considered as statistically significant in the current study.

Secondly, a meta‐analysis with GEO and ArrayExpress data was performed with stata v. 12.0 (StataCorp, College Station, TX, USA) and the metan package. Continuous outcomes were presented as standard mean difference (SMD) with 95% confidence interval (CI), and effect sizes were pooled with a random or fixed‐effects model based on different conditions. Heterogeneity across studies was assessed with the chi‐square test of *Q* and the *I* ^2^ statistic. A *P* value < 0.05 or *I* ^2^ > 50% was considered heterogeneous. If so, the random‐effects model (the DerSimonian–Laird method) would be selected to calculate the summarized SMD. If not, the fixed‐effects model (the Mantel–Haenszel method) was preferred for the pooling process.

If heterogeneity was present, to further explain whether the pooled result was achieved due to one large study or a single study with an extremely divergent result, sensitivity analysis was applied to omit one study at a time. In addition, the potential publication bias was evaluated with Begg's and Egger's tests. If *P* < 0.05, there would be publication bias.

Another approach, meta‐analysis with summary receiver operating characteristic (SROC), was further carried out to verify the expression level of miR‐193a‐3p in NSCLC cases. Further, diagnostic odds ratio analysis was executed to assess the diagnostic possibility of miR‐193a‐3p for NSCLC patients. Positive and negative likelihood ratios were also obtained to reflect the diagnostic value of miR‐193a‐3p.

### Implication of miR‐193a‐3p expression in NSCLC based on The Cancer Genome Atlas data

The Cancer Genome Atlas (TCGA) only offered expression data of precursor miRNA; therefore, we extracted the miR‐193a expression data from lung adenocarcinoma (LUAD) and lung squamous cell carcinoma (LUSC) patients. We also collected the clinical pathological parameters of the patients. The differences of miR‐193a between different groups were assessed as mentioned above. The association between miR‐193a expression and the clinicopathological features was determined using spss statistics 23.0.

### Meta‐analysis of studies from literatures

PubMed, Web of Science, EMBASE, Science Direct, Wiley Online Library, Ovid, Cochrane Central Register of Controlled Trials, LILACs and Google Scholar, and the CNKI, VIP, CBM and WanFang databases were used to search for studies evaluating the clinical value of miR‐193a‐3p in NSCLC. Literature published up to April 2017 was retrieved. The search terms were the same as aforementioned for the GEO database. All eligible studies were included in line with the following criteria: (a) NSCLC patients should be affirmed pathologically; (b) the expression level of miR‐193a‐3p should be evaluated for NSCLC patients or mean and standard deviation (SD) should be provided; (c) the literature should be the most complete or recent if the same patient cohort was reported more than once by the same authors or research group; (d) it should be written in either Chinese or English in full text.

### Meta‐analysis based on GEO and TCGA databases and literature

To obtain the comprehensive picture of the clinical role of miR‐193a‐3p in NSCLC, we combined all available data from GEO, TCGA and literature in the final meta‐analyses. Similar approaches were performed as aforementioned, including the calculation of SMD and SROC.

## Potential function inquiry of miR‐193a‐3p in NSCLC

### Identification of prospective target genes of miR‐193a‐3p

To gain possible target genes of miR‐193a‐3p, 12 online target gene prediction databases were used, namely miRBase, miRDB, miRWalk, RNA22, Targetscan, miR.org, Tarbase, mirTarBase, PicTar‐vet, Targetminer, PITA and PolymiRTS. Genes overlapping in more than five databases were collected as the ‘prediction set’ of miR‐193a‐3p target genes. At the same time, verified genes were gained from Tarbase, mirTarBase, miRWalk2.0, miRecords and literature.

In addition, we assessed the differentially expressed genes of LUAD and LUSC from TCGA. All datasets were processed and calculated for total read counts and reads per million values. The edger package was used to perform the statistical analysis. All the dysregulated genes of LUAD and LUSC were obtained for intersection elements. Finally, the overlap section of predicted target genes and the dysregulated ones from TCGA were collected for the following analysis.

### Signaling pathway and gene network analyses

The potential target genes of miR‐193a‐3p achieved above were pooled for further GO and KEGG analyses with Database for Annotation, Visualization and Integrated Discovery (DAVID), which was applied to perform GO enrichment and KEGG pathway analysis. The predicted target genes were uploaded to DAVID, and only pathways with *P* < 0.05 were considered to be statistically significant. The STRING database was utilized to construct the PPI network for the hub gene identification. Hub genes were regarded as the key target genes of miR‐193a‐3p in NSCLC. Associations among proteins were assessed by adopting a confidence score threshold of > 0.4.

## Results

### Features of the included datasets from GEO and ArrayExpress

Altogether, 19 eligible datasets were involved in the meta‐analysis, including GSE17681 (Germany), GSE27486 (USA), GSE31586 (Germany), GSE40738 (USA), GSE46729 (USA), GSE61741 (Germany), GSE68951 (Germany), GSE14936 (USA), GSE16025 (USA), GSE15008 (China), GSE25508 (Finland), GSE19945 (Japan), GSE47525 (Netherlands), GSE48414 (Norway), GSE51853 (Japan), GSE63805> (USA), GSE 72526 (Switzerland), GSE74190 (China) and GSE77380 (Japan).

The data for GSE17681 (Germany), GSE27486 (USA), GSE31586 (Germany), GSE40738 (USA) and GSE61741 (Germany) were derived from peripheral blood, and the data for GSE46729 and GSE68951 were from serum and plasma, respectively. The data for GSE14936 (USA), GSE16025 (USA), GSE15008 (China), GSE25508 (Finland), GSE19945 (Japan), GSE47525 (Netherlands), GSE48414 (Norway), GSE51853 (Japan), GSE63805 (USA), GSE72526 (Switzerland), GSE74190 (China) and GSE77380 (Japan) were from NSCLC tissues.

In total, 453 cases of NSCLC patients and 476 normal controls were contained in blood samples, while 920 cases of NSCLC patients and 406 normal controls provided tissue samples (Table 1).

**Table 1:** Characteristics of hsa‐miR‐193a‐3p expression profiling datasets included in the current meta‐analysis. NSCLC, non‐small cell lung carcinoma; SCLC, small cell lung carcinoma

Series	Country	Sample type	Platform	Lung cancer types	Sample of lung cancer patients	Sample of healthy controls	Citation (ref.)	Year
GSE61741	Germany	Peripheral blood	GPL9040	NSCLC	73	94	Keller A	2014
GSE46729	USA	Serum	GPL8786	NSCLC	24	24	Godrey A, *et al*.	2014
GSE40738	USA	Whole blood	GPL16016	NSCLC	82	58	Patnaik SK, *et al*.	2012
GSE27486	USA	Whole blood	GPL11432	NSCLC^a^	22	23	Patnaik SK, *et al*.	2011
GSE68951	Germany	Plasma	GPL16770	NSCLC	203	12	Leidinger P, *et al*.	2015
GSE17681	Germany	Peripheral blood	GPL9040	NSCLC	17	198	Keller A, *et al*.	2009
GSE31568	Germany	Peripheral blood	GPL9040	NSCLC	32	67	Keller A, *et al*.	2011
GSE14936	USA	Tissue	GPL8879	NSCLC	27	28	Seike M, *et al*.	2009
GSE15008	China	Tissue	GPL8176	NSCLC	182	185	Tan X, *et al*.	2010
GSE16025	USA	Tissue	GPL5106	NSCLC	58	5	Raponi M, *et al*.	2009
GSE19945	Japan	Tissue	GPL9948	NSCLC, SCLC	55	8	Ohba T, *et al*.	2013
GSE25508	Finland	Tissue	GPL7731	NSCLC	26	26	Guled M, *et al*.	2011
GSE47525	Netherlands	Tissue	GPL17222	NSCLC	18	14	van Jaarsveld MT, *et al*.	2013
GSE48414	Norway	Tissue	GPL16770	NSCLC	154	20	Bjaanaes MM, *et al*.	2014
GSE51853	Japan	Tissue	GPL7341	NSCLC	124	5	Arima C, *et al*.	2014
GSE63805	USA	Tissue	GPL18410	NSCLC	32	30	Robles AI, *et al*.	2015
GSE72526	Switzerland	Tissue	GPL20275	NSCLC	67	18	Gasparini P, *et al*.	2015
GSE74190	China	Tissue	GPL19622	NSCLC	92	44	Jin Y, *et al*.	2015
GSE77380	Japan	Tissue	GPL16770	NSCLC, SCLC	85	23	Yoshimoto T	2016

a Only adenocarcinomas were involved.

### Potential Diagnostic value of miR‐193a‐3p as a marker for NSCLC based on GEO and ArrayExpress

**Figure 1:**
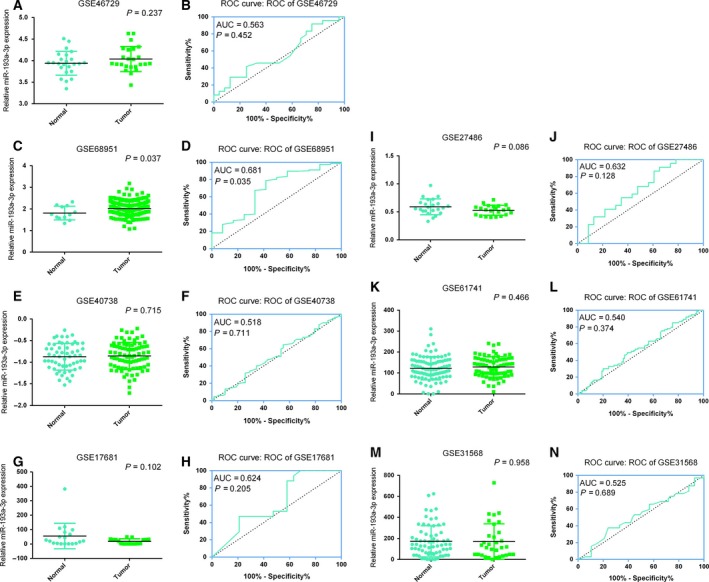
Diagnostic value of miR‐193a‐3p expression in NSCLC patient samples. (A,C,E,G,I,K,M) Plot diagram of miR‐193a‐3p expression based on blood samples. (B,D,F,H,J,L,N)ROC curve of miR‐193a‐3p for diagnosis of NSCLC patients based on blood samples.

**Figure 2:**
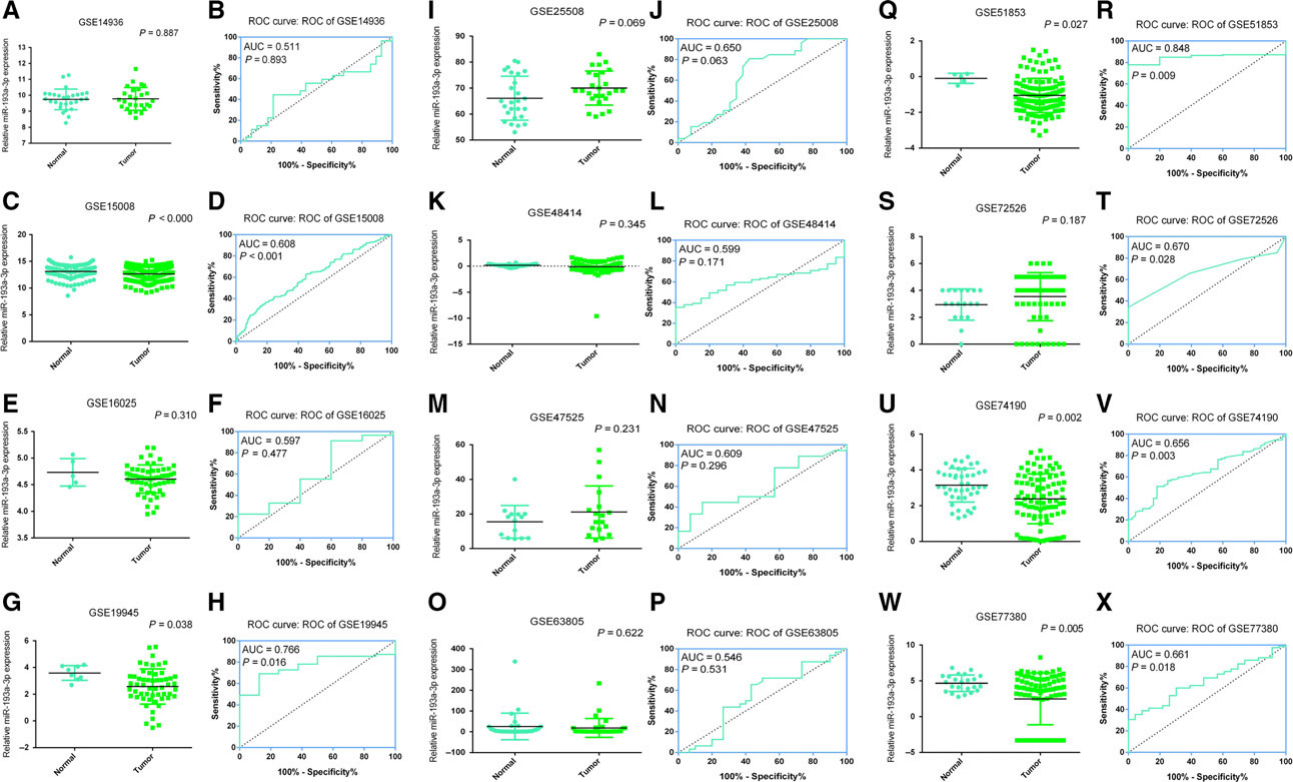
(A,C,E,G,I,K,M,O,Q,S,U,W) Plot diagram of miR‐193a‐3p expression based on tissue samples. (B,D,F,H,J,L,N,P,R,T,V,X) ROC curve of miR‐193a‐3p for diagnosis of NSCLC patients based on tissue samples.

Firstly, the expression level of miR‐193a‐3p was extracted from each microarray. Student's paired or unpaired *t* test was used to assess the alteration of miR‐193a‐3p level between different groups with spss statistics 23.0. ROC curve analyses were used to assess the diagnostic significance of miR‐193a‐3p in NSCLC (Figs 1 and 2). *P*  < 0.05 was regarded as being statistically significant in the current study.

**Figure 3:**
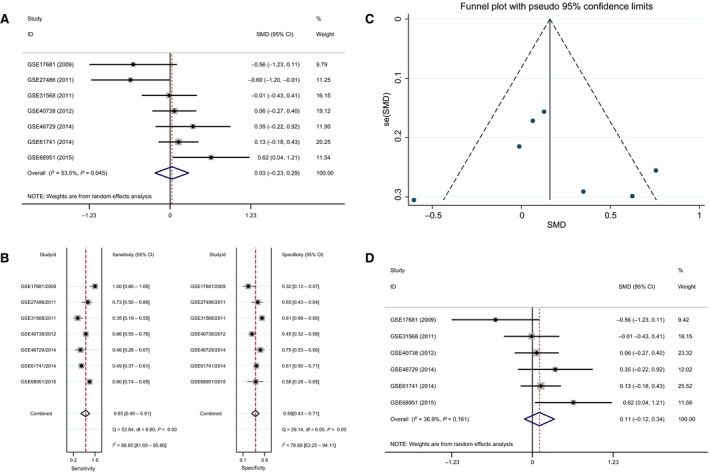
(A) Forest plot of the diagnostic value of blood miR‐193a‐3p in NSCLC. (B) Sensitivity analysis of blood miR‐193a‐3p in NSCLC. (C) Begg's funnel plot of blood miR‐193a‐3p in NSCLC; (D) Forest plot of the diagnostic value of blood miR‐193a‐3p in NSCLC after removing GSE25486.

In the meta‐analysis of blood samples, the SMD ranged from −0.56 to 0.62 among the seven datasets (Fig. 3A). According to the result of the heterogeneity test, there was significant heterogeneity in these datasets (*P* = 0.045, *I* ^2^ = 53.5%). Thus, the expression level of blood miR‐193a‐3p between the NSCLC and normal controls was of no difference based on the random‐effects model (SMD = 0.03; 95% CI, −0.23 to 0.29).

According to sensitivity analysis, results indicated that study GSE27486 had the most negative influence on the summary SMD, which was consistently verified by Begg's funnel plot (Fig. 3B,C). Thus, study GSE27486 was removed and the pooled SMD changed to 0.11 (95% CI, ‐0.12 to 0.34) as assessed by the random‐effects model, since the heterogeneity was still available (*P* = 0.161, *I* ^2^ = 36.9%, Fig. 3D).

**Figure 4:**
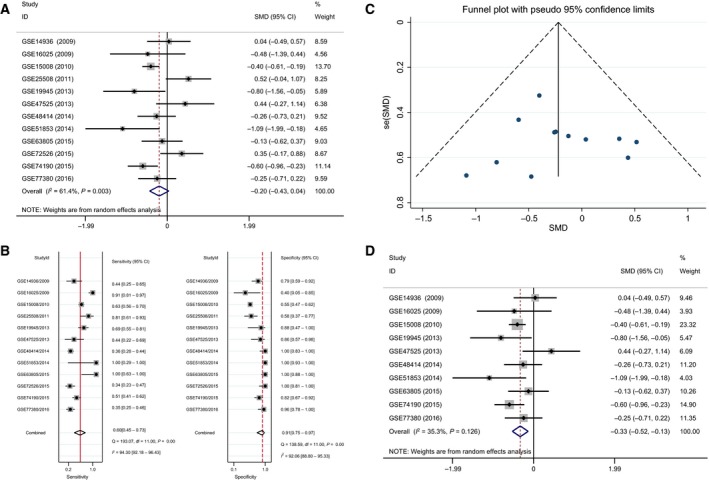
(A) Forest plot of the diagnostic value of tissue miR‐193a‐3p in NSCLC. (B) Sensitivity analysis of tissue miR‐193a‐3p in NSCLC. (C) Begg's funnel plot of tissue miR‐193a‐3p in NSCLC. (D) Forest plot of the diagnostic value of tissue miR‐193a‐3p in NSCLC after removing GSE72526 and GSE25508.

In the meta‐analysis for tissue samples, the SMD ranged from ‐1.09 to 0.52 among the 12 datasets (Fig. 4A). According to the result of the heterogeneity test, there was significant heterogeneity in these datasets (*P* = 0.003, *I* ^2^ = 61.4%). Thus, the expression of tissue miR‐193a‐3p had no difference between the NSCLC and normal controls based on the random‐effects model (SMD = −0.20; 95% CI, −0.43 to 0.04).

The results of the sensitivity analysis indicated that the microarrays of GSE72526 and GSE25508 had the most negative influence on the summary SMD, which was consistently verified by Begg's funnel plot (Fig. 4B,C). Thus, the studies GSE72526 and GSE25508 were removed and the pooled SMD changed to ‐0.33 (95% CI, ‐0.52 to ‐0.13) as assessed by the random‐effects model, since the heterogeneity was still present (*P* = 0.126, *I* ^2^ = 35.3%, Fig. 4D).

**Figure 5:**
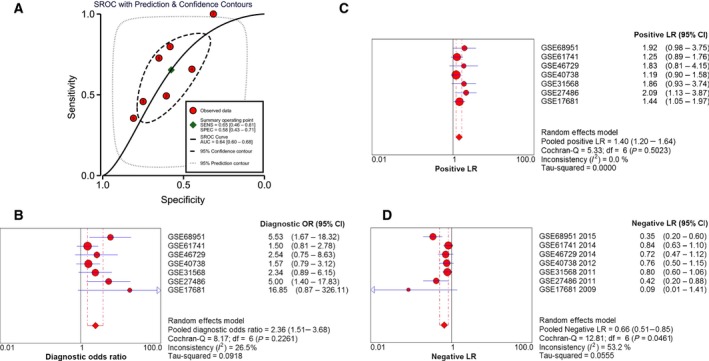
(A) SROC curve of blood mir‐193a‐3p. The area under curve (AUC) of miR‐193a‐3p was 0.64 (95% CI, 0.60–0.68). (B) Diagnostic odds ratio of blood mir‐193a‐3p. (C) Positive LR of blood mir‐193a‐3p. (D) Negative LR of blood mir‐193a‐3p.

In addition, a SROC curve was plotted to show the diagnostic implications of miR‐193a‐3p in NSCLC. Firstly, in the samples of blood, the AUC of the SROC curve was 0.64 (Fig. 5A), which indicated that blood miR‐193a‐3p played a vital part in the early diagnosis of NSCLC. The pooled diagnostic odds ratio 2.36 (95% CI, 1.51–3.68, *P* = 0.226, Fig. 5B) also proved that the expression level of miR‐193a‐3p could distinguish patients from healthy people, which was verified by the pooled positive LR 1.40 (95% CI, 1.20–1.64, *P* = 0.502, Fig. 5C) and the pooled negative LR 0.66 (95% CI, 0.51–0.85, *P* = 0.046, Fig. 5D).

**Figure 6:**
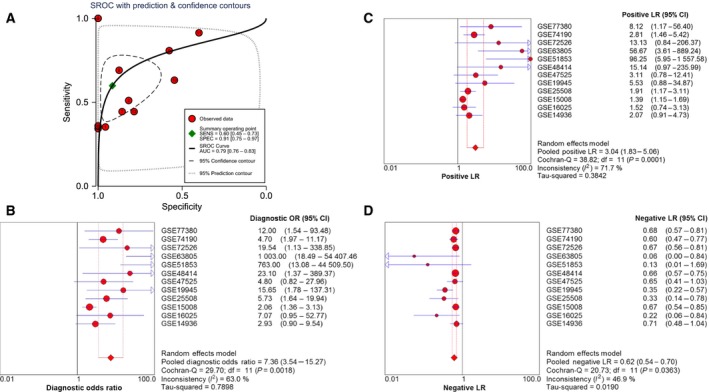
(A) SROC curve of tissue miR‐193a‐3p. The area under curve (AUC) of miR‐193a‐3p was 0.79 (95% CI, 0.76–0.83). (B) Diagnostic odds ratio of tissue miR‐193a‐3p. (C) Positive LR of tissue mir‐193a‐3p. (D) Negative LR of tissue miR‐193a‐3p.

In the samples of tissue, the AUC of the SROC curve was 0.79 (Fig. 6A), which indicated that the diagnostic ability of miR‐193a‐3p of tissue was more obvious than that of blood. The pooled diagnostic odds ratio 7.36 (95% CI, 3.54–15.27, *P* = 0.0018, Fig. 6B) also proved that the expression level of miR‐193a‐3p could distinguish patients from healthy people, which was verified by the pooled positive LR 3.04 (95% CI, 1.83–5.06, *P* < 0.001, Fig. 6C) and the pooled negative LR 0.62 (95% CI, 0.54–0.70, *P* = 0.036, Fig. 6D).

### Characteristics of the patients with NSCLC from TCGA

The correlation between the expression of miR‐193a and clinic pathological features in LUAD and LUSC tissues from TCGA were enumerated. Significant differences of miR‐193a expression level was found between lung cancer tissues and adjacent non‐cancerous ones in LUSC (*P* < 0.001). The expression of miR‐193a was lower in lung cancer when compared with adjacent non‐cancerous lung. LUSC patients with a smoking habit (7.66 ± 0.79) had greater up‐expression of miR‐193a than those without the habit (7.45 ± 0.88, *P* = 0.023, Tables 2, 3, 4).

**Table 2:** Relationship between the expression of miR‐193a and clinicopathological features in LUSC from TCGA (mean ± SD). For RPKM, Student's paired or unpaired *t* test was used

Clinicopathological feature		*n*	miR‐193a relevant expression		
			RPKM (log2)	*t*	*P*
Tissue	Lung cancer	480	7.525952 ± 0.865471	−4.948157	0.000004
	Adjacent non‐cancerous lung	45	7.888973 ± 0.414712		
Age	> 65	185	7.525579 ± 0.934945	0.050481	0.959761
	≤ 65	289	7.521452 ± 0.823057		
Gender	Male	347	7.558295 ± 0.865309	1.637792	0.102140
	Female	121	7.408978 ± 0.858448		
Smoker	Yes	130	7.656648 ± 0.790222	2.277028	0.023243
	No	332	7.454980 ± 0.880344		
Clinical TNM stage	I and II	378	7.521731 ± 0.846972	0.254974	0.798856
	III and IV	88	7.495593 ± 0.944560		
T	T1 and T2	380	7.484478 ± 0.855391	−1.834394	0.067233
	T3 and T4	88	7.671741 ± 0.894978		
N	Yes	167	7.421628 ± 0.937902	−1.652229	0.099505
	No	295	7.565224 ± 0.821211		
M	Yes	6	8.014767 ± 0.412640	1.541487	0.124018
	No	382	7.458407 ± 0.881689		
Tumor diameter (cm)	> 0.9 cm	244	7.496234 ± 0.783197	−0.689266	0.491016
	≤ 0.9 cm	230	7.551525 ± 0.949650		
Location	Central	136	7.585938 ± 0.831344	−0.437418	0.662227
	Peripheral	91	7.636411 ± 0.882065		
Residual tumor	Yes	14	7.627271 ± 0.597706	0.708142	0.479285
	No	374	7.455612 ± 0.898980		
Neoadjuvant treatment	Yes	6	7.213383 ± 0.602037	−0.872681	0.383287
	No	462	7.523668 ± 0.867740		
Radiation therapy	Yes	41	7.486063 ± 0.787671	−0.300110	0.764266
	No	321	7.527955 ± 0.848168		
Targeted molecular therapy	Yes	113	7.451135 ± 0.797590	−0.998845	0.318533
	No	253	7.545861 ± 0.855592		

**Table 3:** Univariate Cox regression analysis of characteristics in LUSC. B, B value is the regression coefficient and intercept (constant term); HR, hazard ratio; SE, standard error; Sig., *P* value

Characteristic	B	SE	Wald statistic	df	Sig.	HR	95% CI
Clinical TNM stage	0.472445	0.175108	7.279332	1	0.006975	1.603911	1.137963–2.260643
T	0.547240	0.179851	9.258232	1	0.002344	1.728476	1.214992–2.458970
N	−0.254714	0.149093	2.918706	1	0.087558	0.775138	0.578723–1.038215
M	−0.273135	0.099921	7.472106	1	0.006266	0.760990	0.625643–0.925618
Tumor diameter (cm)	0.117830	0.152824	0.594470	1	0.440696	1.125053	0.833852–1.517947
Residual tumor	−0.181177	0.102099	3.148928	1	0.075977	0.834288	0.682982–1.019114
Neoadjuvant treatment	−0.024768	0.583556	0.001801	1	0.966145	0.975536	0.310827–3.061734
Radiation therapy	−0.133596	0.252688	0.279522	1	0.597014	0.874944	0.533202–1.435716
Targeted molecular therapy	0.430070	0.209421	4.217337	1	0.040013	1.537365	1.019806–2.317588

**Table 4:** Multivariate Cox regression analysis of characteristics in LUSC. B, B value is the regression coefficient and intercept (constant term); HR, hazard ratio; SE, standard error; Sig., *P* value

Characteristic	*B*	SE	Wald statistic	df	Sig.	HR	95% CI
T	0.462151	0.244642	3.568666	1	0.058880	1.587485	0.982811–2.564185
M	−0.347973	0.104690	11.047946	1	0.000888	0.706118	0.575129–0.866941
Clinical TNM stage	0.285211	0.250161	1.299845	1	0.254242	1.330043	0.814569–2.171718
Targeted molecular therapy	0.497774	0.214111	5.404887	1	0.020080	1.645055	1.081257–2.502834

For LUAD, patients with a smoking habit (7.58 ± 0.90) also had greater up‐expression of miR‐193a than those without it (7.36 ± 0.88, *P* = 0.020). Concerning the clinical tumor, node and metastasis (TNM) stage of LUAD, the relative level of miR‐193a was remarkably higher in stage N than in other stages (7.5 ± 0.87, *P* = 0.023). Compared with a peripheral location (7.55 ± 0.98), the expression of miR‐193a was reduced in central locations in LUAD (7.21 ± 0.79, *P* = 0.021, Tables 5, 6, 7).

**Table 5:** Relationship between the expression of miR‐193a and clinicopathological features in LUAD from TCGA (mean ± SD). For RPKM, Student's paired or unpaired *t* test was used

Clinicopathological feature		*n*	miR‐193a relevant expression		
			RPKM (log^2^)	*t*	*P*
Tissue	Lung cancer	513	7.214102 ± 0.787625	−1.918105	0.061056
	Adjacent non‐cancerous lung	46	7.548920 ± 0.981258		
Age	> 65	256	7.368675 ± 0.908801	−0.877582	0.380600
	≤ 65	236	7.438942 ± 0.863317		
Gender	Male	239	7.423370 ± 0.945404	0.135752	0.892071
	Female	274	7.412676 ± 0.838690		
Smoker	Yes	119	7.579224 ± 0.899954	2.326100	0.020416
	No	378	7.362792 ± 0.880509		
Clinical TNM stage	I and II	398	7.380446 ± 0.884425	−1.828929	0.068001
	III and IV	108	7.555999 ± 0.885666		
T	T1 and T2	442	7.391222 ± 0.887205	−1.350914	0.177327
	T3 and T4	66	7.549444 ± 0.889854		
N	Yes	171	7.540150 ± 0.872370	2.286925	0.022620
	No	328	7.349028 ± 0.893038		
M	Yes	23	7.426187 ± 1.201434	0.308948	0.757537
	No	344	7.364119 ± 0.912881		
Tumor diameter (cm)	> 7.4 cm	197	7.485256 ± 0.831706	0.188783	0.850369
	≤ 7.4 cm	165	7.467500 ± 0.957637		
Location	Central	61	7.214102 ± 0.787625	−2.321373	0.021368
	Peripheral	124	7.548920 ± 0.981258		
Residual tumor	Yes	16	7.590387 ± 0.851161	1.268497	0.205456
	No	339	7.365456 ± 0.884371		
Neoadjuvant treatment	Yes	3	7.868600 ± 0.907847	0.882194	0.378088
	No	508	7.414468 ± 0.888921		
Radiation therapy	Yes	48	7.590387 ± 0.851161	1.656416	0.098455
	No	338	7.365456 ± 0.884371		
Targeted molecular therapy	Yes	124	7.459590 ± 0.858687	0.967914	0.333700
	No	260	7.366328 ± 0.894127		

**Table 6:** Univariate Cox regression analysis of characteristics in LUAD. B, B value is the regression coefficient and intercept (constant term); HR, hazard ratio; SE, standard error; Sig., *P* value

Characteristic	B	SE	Wald statistic	df	Sig.	HR	95% CI
Clinical TNM stage	0.983100	0.155107	40.172597	1	0.000000	2.672728	1.972092–3.622284
T	0.716035	0.198479	13.014856	1	0.000309	2.046304	1.386834–3.019368
N	−0.681987	0.119488	32.576217	1	0.000000	0.505612	0.400045–0.639036
M	0.022253	0.087000	0.065424	1	0.798120	1.022502	0.862204–1.212603
Tumor diameter (cm)	0.053987	0.179151	0.090811	1	0.763148	1.055471	0.742938–1.499477
Residual tumor	−1.354798	0.308974	19.226680	1	0.000012	0.257999	0.140805–0.472735
Neoadjuvant treatment	−0.567799	0.273796	4.300646	1	0.038098	0.566772	0.331400–0.969313
Radiation therapy	−0.727292	0.219748	10.953925	1	0.000934	0.483216	0.314117–0.743345
Targeted molecular therapy	−0.363251	0.186343	3.800016	1	0.051252	0.695412	0.482643–1.001977

**Table 7:** Multivariate Cox regression analysis of characteristics in LUAD. B, B value is the regression coefficient and intercept (constant term); HR, hazard ratio; SE, standard error; Sig., *P* value

Characteristic	B	SE	Wald statistic	df	Sig.	HR	95% CI
T	0.576365	0.295382	3.807376	1	0.051027	1.779558	0.997429–3.174988
N	−0.431418	0.207871	4.307350	1	0.037948	0.649587	0.432213–0.976287
Clinical TNM stage	0.586918	0.207871	5.695250	1	0.017011	1.798437	1.110592–2.912299
Residual tumor	−0.921665	0.408787	5.083375	1	0.024156	0.397856	0.178552–0.886515
Neoadjuvant treatment	−2.635720	0.776167	11.531550	1	0.000684	0.071667	0.015655–0.328092
Targeted molecular therapy	0.341323	0.269954	1.598646	1	0.206095	1.406808	0.828800–2.387921
Radiation therapy	−0.684721	0.307561	4.956402	1	0.025994	0.504231	0.275952–0.921351

However, no obvious association was observed between miR‐193a level and other clinicopathological features.

### Information from studies from literature

A total of 17 documents were retrieved, but only one of them met our criteria. Therefore, a meta‐analysis of studies could not be performed. The expression level of miR‐193a‐3p of NSCLC was prominently up‐regulated over that of adjacent non‐cancerous lung tissues (*P* < 0.001) 17.

### Target gene aggregation of miR‐193a‐3p

A total of 512 genes were derived from miRWalk2.0, Tarbase, miRTarbase and miRecords, which were validated by qPCR, western blot or luciferase assay. Twelve online databases were used for the prediction and a total of 2586 targets genes overlapped in at least five databases were obtained. In addition, together with the extracted genes from the literature, we collected a total of 3121 predicted target genes.

From TCGA, 6138 up‐regulated genes in LUAD and LUSC were obtained. Finally, bioinformatics analyses were performed on the 379 overlapping sets of these genes.

### GO and pathway enrichment analyses

**Figure 7:**
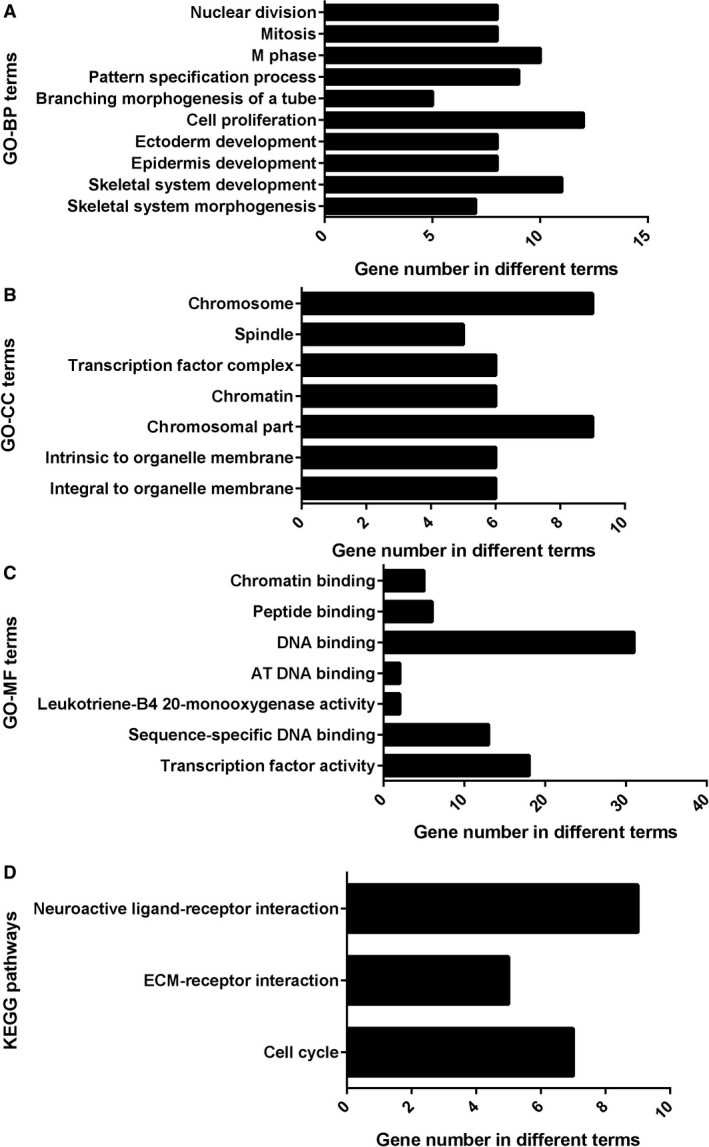
Enriched Gene Ontology (GO) items of overlapping target genes of miR‐193a‐3p. (A) Pathways of biological processes (BP). (B) Pathways of cellular components (CC). (C) Network analysis of molecular function (MF). (D) Kyoto Encyclopedia of Genes and Genomes (KEGG) pathway enrichment analysis of miR‐193a‐3p target genes. The lengths of the bars represent the number of overlapping genes in GO and KEGG. AT, Oligo (A) and oligo (T) tracts of DNA; ECM, extracellular matrix.

**Figure 8:**
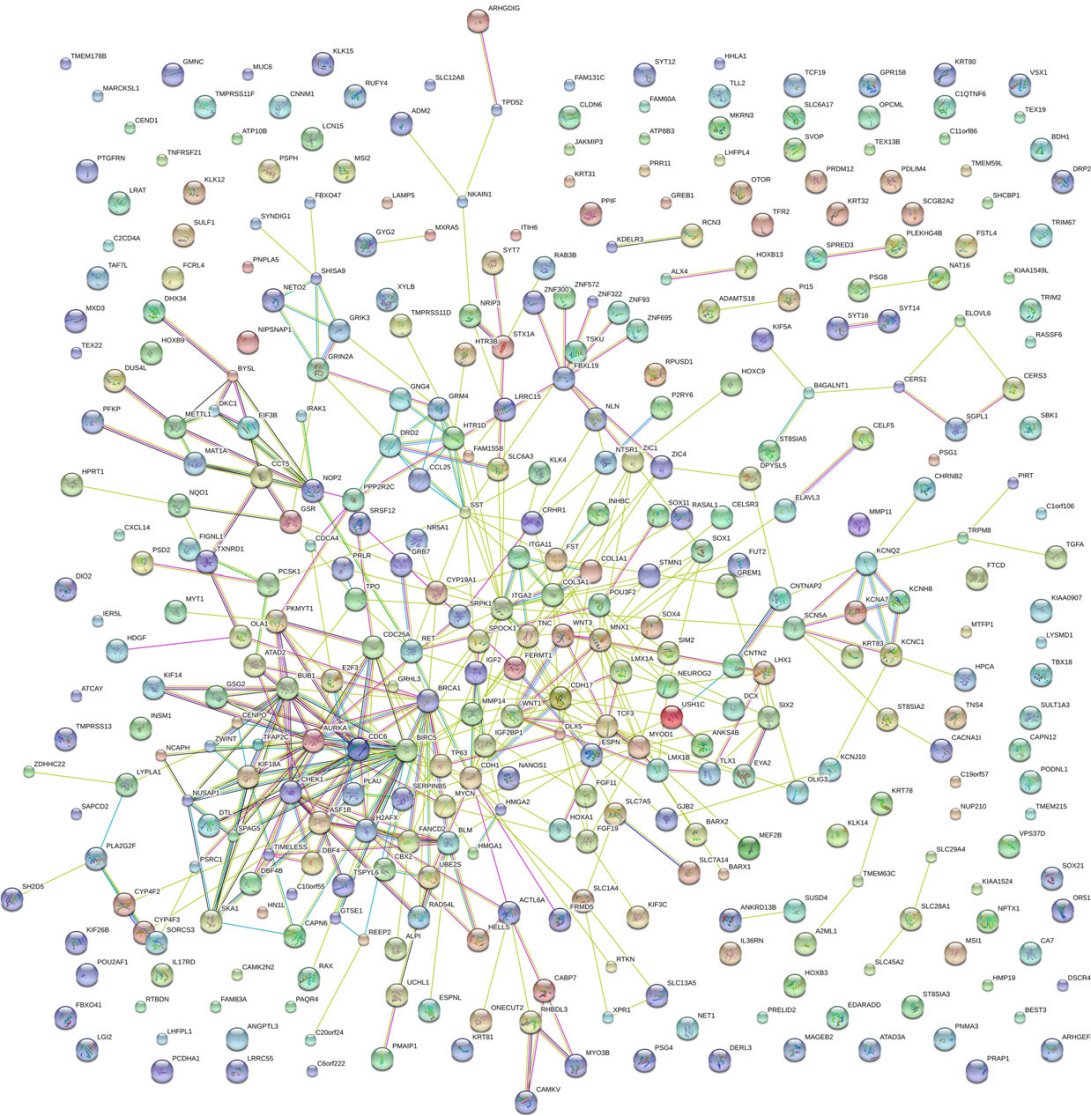
Protein–protein interaction network of all 379 potential genes targeted by miR‐193a‐3p in NSCLC.

**Figure 9:**
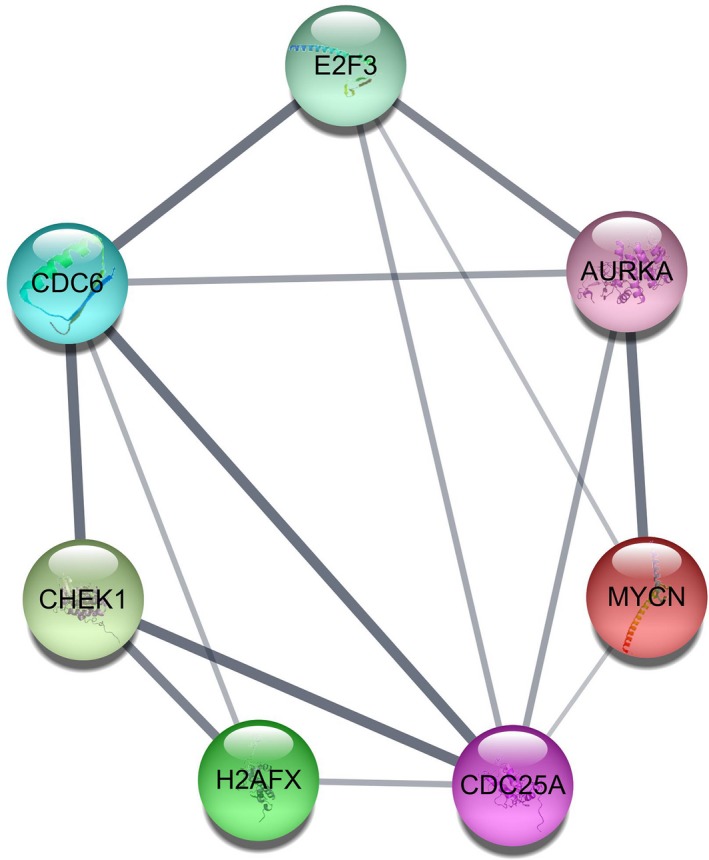
The top seven hub genes of miR‐193a‐3p with the largest number of connections in the protein–protein interaction target gene network.

According to the target‐GO analysis in DAVID, genes were highly concentrated in the biological processes of neurotransmitter transport, skeletal system development, ion transport, etc. (*P* < 0.005, Fig. 7A), in the cellular components of integral to organelle membrane, intrinsic to organelle membrane, chromosomal part, etc. (*P* < 0.05, Fig. 7B), and in the molecular functions of transcription factor activity, sequence‐specific DNA binding, leukotriene‐B4 20‐monooxygenase activity, etc. (*P* < 0.05 Fig. 7C). In KEGG pathway analysis, target genes mainly gathered at pathways of cell cycle (*P* < 0.05, Fig. 7D). In addition, we conducted protein–protein interaction (PPI) by STRING 10.0 to find the hub genes of mir‐193a‐3p. *E2F3*,* CDC6*,* AURKA*,* CHEK1*,* H2AFX*,* CDC25A* and *MYCN* were obtained (Figs 8 and 9).

## Discussion

miRNAs have the potential to function as steady and reproducible biomarkers for different solid malignant tumors, especially NSCLC 23, 24. The methylation of the mir‐193a gene has an indirect effect on the expression level of the target gene 25, 26, 27. In the oral squamous cell carcinoma, the miR‐193a gene is hypermethylated and the expression of miR‐193a was frequently down‐regulated 26. Conversely, miR‐193a gene methylation silencing could reduce miR‐193a expression in some diseases, for example acute myeloid leukemia and NSCLC 27, 28. Downregulation of the miR‐193a level in NSCLC tissues was reported by Chen *et al*. 29. In addition, our previous study found that the miR‐193a‐3p level was reduced in NSCLC tissues 17. In the current study, we studied the miR‐193a expression of NSCLC patients from TCGA and their corresponding paracancer tissue (PT). We found lower expression of miR‐193a in NSCLC tissues, in comparison with non‐cancerous controls, which confirmed the findings of Chen *et al*. 29. The relevant level of miR‐193a in LUAD was predominantly down‐regulated compared with that of the PT (*P* < 0.001). Additionally, the relevant level of miR‐193a in LUSC was also less than that of the PT (*P* = 0.0056). The additional ROC curve demonstrated that miR‐193a had a diagnostic value for NSCLC, especially for LUAD. Thus, we were strongly convinced that miR‐193a could be a tumor‐suppressive predictor in NSCLC.

Since few studies have concentrated on the correlation between the expression of miR‐193a and clinic pathological features of NSCLC, we recruited patients to investigate the correlation between the expression of miR‐193a and clinicopathological features in LUAD and LUSC from TCGA. Firstly, there was a significant alteration of relevant miR‐193a expression between lung cancer and adjacent non‐cancerous lung. miR‐193a was down‐regulated in lung cancer compared with PT, which suggested its role in diagnosis. Secondly, the smoking habit was the common risk factor for LUAD and LUSC. However, there was a significant difference between miR‐193a and location and clinical TNM stage in LUAD. Tumors located in central parts expressed lower miR‐193a than those in peripheral parts. As for clinical TNM stage, miR‐193a was expressed at high levels in LUAD patients whose tumors were in stage N. These two indexes indicated poor prognosis and metastasis of LUAD. As for the discrepancy of miR‐193a expression in LUSC and LUAD, the highly expressed miR‐193a in the peripheral location and stage N of LUAD might be due to tumor cell differentiation. The exact mechanism needs further investigation.

miR‐193a‐3p is a member in the miR‐193a family. Nonetheless, information for miR‐193a‐3p is inadequate and its molecular function and mechanism in early diagnosis remain unidentified. No specific blood miRNA has been verified as a biomarker in the clinic for the early screening of NSCLC. Only two studies have explored the diagnostic value of miR‐193a‐3p, in hepatocellular carcinoma and colorectal cancer 15, 30. miR‐193a‐3p attracted our attention and in the present study we attempted to investigate the potential value of miR‐193a‐3p in early screening of NSCLC based on microarray databases, and to further explore its prospective relevant pathways via bioinformatics analysis.

In the meta‐analysis, a total of seven blood miR‐193a‐3p microarray datasets were involved, including 453 NSCLC patients and 297 healthy controls. The random effects model showed that significant inconsistency of miR‐193a‐3p expression was noticeable between NSCLC patients and healthy controls, since the AUC of the SROC curve was 0.64, which indicated a diagnostic value of blood miR‐193a‐3p in NSCLC compared with non‐cancerous controls.

Since we found a significantly lower miR‐193a‐3p level in NSCLC tissues, down‐regulation of miR‐193a‐3p also played a vital role in the prognosis of NLCLC 17. In the following study, we attempted to probe the probable value for tissue miR‐193a‐3p. In sum, we studied 12 tissue miR‐193a‐3p microarray datasets including 920 NSCLC patients and 406 healthy controls. The pooled diagnostic odds ratio was 7.36 (95% CI, 3.54–15.27, *P* = 0.0018). In addition, the AUC of the SROC curve was 0.79, which suggested that tissue miR‐193a‐3p had a reliable diagnostic value for NSCLC. Thus, we were strongly convinced that tissue miR‐193a‐3p was a tumor‐suppressive predictor in NSCLC. However, the diagnostic value of tissue miR‐193a‐3p still requires more studies to confirm this.

Concerning the molecular mechanism of miR‐193a‐3p in NSCLC, several reports have stated the function and molecular mechanism. miR‐193a‐3p could inhibit the metastasis of lung cancer cells by modulating the expression of cancer‐related proteins 31. It could also overpower the metastasis of human NSCLC by suppressing the Erb‐B2 Receptor Tyrosine Kinase 4 (ERBB4)/S6 kinase 2 (S6K2) signaling pathway 19. In addition, our previous study showed that astrocyte elevatedgene‐1 (AEG‐1) had the potential to be one target of miR‐193a‐3p 4. Since bioinformatics analysis might help in understanding the potential molecular mechanism of miR‐193a‐3p in the carcinogenesis and progression of NSCLC, we performed *in silico* predictions to gather all prospective target genes. Finally, we combined the validated genes and prediction genes together and achieved hub genes for miR‐193a‐3p. The GO and KEGG pathway analyses revealed that miR‐193a‐3p could be related to several key signaling pathways of NSCLC, such as modulation of apoptosis and modulation of programmed cell death in biological processes; organelle lumen, membrane‐enclosed lumen and intracellular organelle lumen in cellular components; identical protein binding, enzyme binding and transcription factor binding activities in molecular functions. Also, several vital pathways were illustrated by KEGG enrichment analysis, including pathways of cancer and focal adhesion signaling pathways. PPI showed the hub genes of mir‐193a‐3p, including *E2F3*,* CDC6*,* CHEK1*,* H2AFX*,* CDC25A*,* MYCN* and *AURKA*.

In view of the results we had achieved, we consulted literature to verify our findings. *E2F3* mRNA levels were significantly higher in lung cancer patients in comparison with non‐cancerous lung tissues and its overexpression was related to poor prognosis 32, 33. Also, *CDC6* has been confirmed to be linked to DNA replication to regulate the occurrence and development of lung tumor 34, 35. Overexpression of *CHEK1* in lung cancers was related to poor overall survival 36. An increase in *CDC25A* expression by means of a decrease in miR‐184 promoted cell invasive capacity 37. *MYCN* was found to be overexpressed in NSCLC, which was positively related to a more invasive tumor phenotype and poorer outcome 38. *AURKA* functioned as an oncogene, and its low expression level inhibited tumor cell proliferation, promoted apoptosis and hindered cell cycle development in NSCLC 39, 40.

However, there are some limitations to the current study. Firstly, the sample size in our study was still small, which would weaken the conclusion of the impact of miR‐193a‐3p in NSCLC patients. Secondly, the small sample size restricted the reliability of the meta‐analysis. Further larger studies should be conducted to support the under‐expression of tissue miR‐193a‐3p expression in NSCLC. Thirdly, extra bias might be due to the limited regions involved in the current meta‐analysis, since the only blood mir‐193a‐3p datasets were from USA and Germany. Fourthly, the potential target genes were achieved via *in silico* prediction, and further validation will also be needed.

Overall, the current observations showed that significant down‐expression of tissue miR‐193a‐3p was detected in NSCLC patients, which indicated tissue miR‐193a‐3p detection might play an integral part in the early diagnosis of NSCLC compared with blood miR‐193a‐3p. Furthermore, the pathway analyses of the prospective target genes of miR‐193a‐3p revealed several key signaling pathways correlated with the incidence and worsening of NSCLC, including the hub genes, such as neuroactive ligand–receptor interaction, cell cycle, etc. Cohorts with larger sample sizes and further *in vitro* and *in vivo* studies are needed to determine the diagnostic value and relevant mechanism of tissue miR‐193a‐3p in NSCLC.

## Author contributions

XG, RT and GC conceived and designed the experiments. XG, RT, QX, JL, HS, GC and ZL performed the experiments. XG, RT, QX, JL, HS, GC and ZL analyzed the data. XG, RT, QX, JL, HS, GC and ZL contributed reagents/materials/analysis tools. XG, QX and JL wrote the manuscript. All authors read and approved the final manuscript.
